# Ruxolitinib reduces *JAK2* p.V617F allele burden in patients with polycythemia vera enrolled in the RESPONSE study

**DOI:** 10.1007/s00277-017-2994-x

**Published:** 2017-04-30

**Authors:** Alessandro Maria Vannucchi, Srdan Verstovsek, Paola Guglielmelli, Martin Griesshammer, Timothy C. Burn, Ahmad Naim, Dilan Paranagama, Mahtab Marker, Brian Gadbaw, Jean-Jacques Kiladjian

**Affiliations:** 1 Center for Research and Innovation of Myeloproliferative Neoplasms (CRIMM), AOU Careggi, Largo Brambilla 3 - Padiglione 27B, 50134 Florence, Italy; 2 Laboratorio Congiunto and Department of Experimental and Clinical Medicine, University of Florence, Florence, Italy; 3 Division of Cancer Medicine, University of Texas MD Anderson Cancer Center, 1515 Holcombe Blvd, Unit 418, Houston, TX 77030 USA; 4 University Clinic for Hematology, Oncology, Hemostaseology and Palliative Care, Johannes Wesling Medical Center, Hans-Nolte-Straße 1, 32429 Minden, Germany; 5 UKRUB, University of Bochum, Bochum, Germany; 6 Incyte Corporation, 1801 Augustine Cut-Off, Wilmington, DE 19803 USA; 7 Novartis Pharmaceuticals Corporation, One Health Plaza, East Hanover, NJ 07936 USA; 8 Centre d’Investigations Cliniques (INSERM CIC 1427), Hôpital Saint-Louis, Université Paris Diderot, Paris, France

**Keywords:** Allele burden, *JAK2* p.V617F, Polycythemia vera, Ruxolitinib

## Abstract

In patients with polycythemia vera (PV), an elevated *JAK2* p.V617F allele burden is associated with indicators of more severe disease (e.g., leukocytosis, splenomegaly, and increased thrombosis risk); however, correlations between allele burden reductions and clinical benefit in patients with PV have not been extensively evaluated in a randomized trial. This exploratory analysis from the multicenter, open-label, phase 3 Randomized Study of Efficacy and Safety in Polycythemia Vera With JAK Inhibitor INCB018424 Versus Best Supportive Care trial evaluated the long-term effect of ruxolitinib treatment on *JAK2* p.V617F allele burden in patients with PV. Evaluable *JAK2* p.V617F-positive patients randomized to ruxolitinib (*n* = 107) or best available therapy (BAT) who crossed over to ruxolitinib at week 32 (*n* = 97) had consistent *JAK2* p.V617F allele burden reductions throughout the study. At all time points measured (up to weeks 208 [ruxolitinib-randomized] and 176 [ruxolitinib crossover]), mean changes from baseline over time in *JAK2* p.V617F allele burden ranged from −12.2 to −40.0% (ruxolitinib-randomized) and −6.3 to −17.8% (ruxolitinib crossover). Complete or partial molecular response was observed in 3 patients (ruxolitinib-randomized, *n* = 2; ruxolitinib crossover, *n* = 1) and 54 patients (ruxolitinib-randomized, *n* = 33; ruxolitinib crossover, *n* = 20; BAT, *n* = 1), respectively. Among patients treated with interferon as BAT (*n* = 13), the mean maximal reduction in allele burden from baseline was 25.6% after crossover to ruxolitinib versus 6.6% before crossover. Collectively, the data from this exploratory analysis suggest that ruxolitinib treatment for up to 4 years provides progressive reductions in *JAK2* p.V617F allele burden in patients with PV who are resistant to or intolerant of hydroxyurea. The relationship between allele burden changes and clinical outcomes in patients with PV remains unclear.

**Electronic supplementary material:**

The online version of this article (doi:10.1007/s00277-017-2994-x) contains supplementary material, which is available to authorized users.

## Introduction

Polycythemia vera (PV) is a myeloproliferative neoplasm characterized by erythrocytosis and activating somatic mutations in *Janus kinase (JAK) 2* [1]. Downstream JAK/signal transducer and activator of transcription signaling is constitutively activated by the *JAK2* p.V617F mutation, which is present in approximately 95% of patients with PV [2]. Higher levels of *JAK2* p.V617F allele burden are associated with indicators of more severe disease, including leukocytosis, splenomegaly, and increased risk for thrombosis [3]; however, correlations between allele burden reduction and clinical benefit in PV have not been extensively evaluated in a randomized trial.

The Randomized Study of Efficacy and Safety in Polycythemia Vera With JAK Inhibitor INCB018424 Versus Best Supportive Care (RESPONSE) is a global, multicenter, open-label, phase 3 trial comparing the efficacy and safety of the JAK1/JAK2 inhibitor ruxolitinib with best available therapy (BAT) in patients with PV who are resistant to or intolerant of hydroxyurea [4]. Ruxolitinib was superior to BAT in controlling hematocrit (Hct), reducing spleen volume, and improving symptoms in patients with PV. The evaluation of allele burden changes in the RESPONSE trial was a predefined exploratory end point. Protocol-specified analyses demonstrated that ruxolitinib treatment was associated with decreases in *JAK2* p.V617F allele burden. At week 32, patients in the ruxolitinib treatment arm experienced a mean percentage change from baseline in *JAK2* p.V617F allele burden of –12.2%; in comparison, the *JAK2* p.V617F allele burden increased a mean of 1.2% in patients randomized to BAT [4]. At week 80, the percentage change in *JAK2* p.V617F allele burden from baseline was −22.0% among patients originally randomized to ruxolitinib [5]. Among patients who crossed over from BAT to ruxolitinib, the *JAK2* p.V617F allele burden had changed by a mean of −6.7% at 48 weeks after crossover [5]. In the ruxolitinib group, *JAK2* p.V617F allele burden decreased steadily during the study; the maximal mean change from baseline (measured at week 112) was −34.7%.

The objectives of this exploratory analysis of the RESPONSE trial were to evaluate in greater detail the effect of long-term ruxolitinib treatment on *JAK2* p.V617F allele burden, to explore the relationship between allele burden changes and clinical outcomes, and to further characterize the mutational profile of patients with PV.

## Methods

### Study design and patients

The design of the RESPONSE trial (ClinicalTrials.gov identifier, NCT01243944) has been reported in detail elsewhere [4]. Briefly, patients with PV and spleen volume ≥450 cm^3^ who had not undergone prior JAK inhibitor therapy and were resistant to or intolerant of hydroxyurea were randomized 1:1 to ruxolitinib (initial dosage, 10 mg twice daily) or BAT; dose modification was permitted. BAT options included hydroxyurea (at a dose that did not cause unacceptable side effects), interferon (IFN) or pegylated (PEG) IFN, pipobroman, anagrelide, immunomodulators (e.g., lenalidomide, thalidomide), phlebotomy, or no medication. Phosphorus-32, busulfan, and chlorambucil were prohibited. In cases of lack of response or toxicity requiring drug discontinuation, a change of BAT was permitted. Patients also received low-dose aspirin unless its use was medically contraindicated. At week 32, crossover from BAT to ruxolitinib was permitted if the primary end point had not been met; crossover was also permitted after week 32 in cases of disease progression.

The primary end point of the study was the proportion of patients who had both Hct control and a reduction in spleen volume of ≥35% from baseline at week 32. Hct control was defined as phlebotomy ineligibility from weeks 8 to 32 and ≤1 instance of phlebotomy eligibility between randomization and week 8. Patients were considered eligible for phlebotomy if they had a confirmed Hct >45% that was ≥3 percentage points higher than their baseline Hct level or a confirmed Hct >48%, whichever was lower (confirmed 2–14 days after the initial observation).

The study was conducted in accordance with the Declaration of Helsinki. Each participating site’s institutional review board reviewed and approved the study, and all patients provided written informed consent before inclusion in the study.

### Assessment of allele burden

Evaluating changes in *JAK2* p.V617F allele burden was a predefined exploratory objective of the RESPONSE trial. Blood samples for *JAK2* p.V617F allele burden quantitation were drawn from each patient at baseline; at weeks 32, 56, 80, 112, 144, 176, and 208; at the crossover visit, if applicable; and at the end of treatment visit. Genomic DNA samples were isolated from peripheral blood using previously validated methods [6].

This post hoc analysis measured the *JAK2* p.V617F allele burden in peripheral blood with a limit of detection of 1% *JAK2* p.V617F and a lower limit of quantitation (LLOQ) of 2% *JAK2* p.V617F [6]. *JAK2* p.V617F allele burden was defined as the percentage of mutant allele present relative to the total (i.e., wild-type plus mutant). Changes from baseline in *JAK2* p.V617F allele burden among patients randomized to ruxolitinib and those who crossed over from BAT to ruxolitinib were reported up to week 208; for the crossover cohort, baseline allele burden was defined as the last observation before receiving ruxolitinib. Changes from baseline in *JAK2* p.V617F allele burden were also evaluated in subgroups of patients who had and who did not have IFN treatment before study enrollment and in patients randomized to IFN as BAT who crossed over to ruxolitinib.

The *JAK2* p.V617F mutation was detected by real-time PCR or high-resolution melting analysis. Deep sequencing based on the Ion Torrent™ Personal Genome Machine™ (PGM) System (Thermo Fisher Scientific Inc., Waltham, MA, USA) was used to analyze mutations in 22 genes associated with the JAK/STAT pathway (*JAK1*, *JAK2* [other than *JAK2* p.V617F], *JAK3*, *EZH2*, *ASXL1*, *TET2*, *IDH1*, *IDH2*, *CBL*, *SRSF2*, *DNMT3A*, *NFE2*, *SOCS1*, *SOCS2*, *SOCS3*, *SH2B3*, *STAT1*, *STAT3*, *STAT5A*, *STAT5B*, *SF3B1*, and *U2AF1*). Ion Torrent PGM data were aligned against the human genome (version 19) using NextGENe® software 2.3.1 (SoftGenetics, LLC, State College, PA, USA). Mutations were confirmed using conventional sequencing methods. Mutations in *CALR* were analyzed using high-resolution capillary electrophoresis and confirmed with Sanger sequencing.

Complete molecular response (CMR) and partial molecular response (PMR) were defined using the International Working Group–Myeloproliferative Neoplasms Research and Treatment/European LeukemiaNet consensus criteria [7]. CMR was defined as reduction of the *JAK2* p.V617F allele burden to below the LLOQ for patients who had an allele burden above the LLOQ at baseline (i.e., a 100% reduction in allele burden from baseline). PMR was defined as achieving ≥50% reduction in *JAK2* p.V617F allele burden for patients who had ≥20% allele burden at baseline.

Potential associations with clinical outcomes were evaluated by analyzing reductions in the *JAK2* p.V617F allele burden and relationships to changes in spleen volume, Hct levels, white blood cell counts, and platelet counts at last observation. These evaluations were also conducted in subgroups of patients who experienced a *JAK2* p.V617F allele burden reduction <20 versus ≥20%.

### Statistics

This was an exploratory analysis of *JAK2* p.V617F allele burden assessments obtained during the RESPONSE trial and was not designed to address specific hypotheses related to *JAK2* p.V617F allele burden or other biomarkers. All data in the current analysis were summarized descriptively.

## Results

### Patients and baseline characteristics

Baseline *JAK2* p.V617F status data were available for 215 of 222 patients initially randomized in the RESPONSE trial. Of 215 patients with baseline *JAK2* p.V617F allele assessments, 104 of 107 patients initially randomized to ruxolitinib and 107 of 108 patients randomized to BAT were *JAK2* p.V617F-positive (Table 1). Ninety-seven patients in the BAT arm crossed over to ruxolitinib and were evaluable for *JAK2* p.V617F allele burden. Median (range) time to crossover in these patients was 34.0 weeks (31.0–53.1). The mean (SD) treatment duration was 25 months (10) and 8 months (2) for patients originally randomized to ruxolitinib and BAT, respectively. Of the patients who received IFN treatment before randomization (ruxolitinib arm, *n* = 18; BAT arm, *n* = 16), the majority (*n* = 11 and *n* = 15, respectively) received non-PEG IFN only.

**Table 1:** Demographics and baseline characteristics for patients with baseline *JAK2* p.V617F assessment

	Ruxolitinib (*n* = 107)	BAT (*n* = 108)
*JAK2* p.V617F mutation-positive, *n* (%)	104 (97.2)	107 (99.1)
*JAK2* p.V617F allele burden^a^, median (range), %	82.5 (10.0–96.0)	84.0 (6.0–97.0)
Median (range) age (years)	62 (34–90)	60 (33–84)
Men, *n* (%)	64 (59.8)	77 (71.3)
Median (range) duration of disease (months)	101 (6–427)	116 (7–271)
Mean (SD) treatment duration (months)	25 (10)	8 (2)
Prior IFN treatment, *n* (%)	18 (16.8)	16 (14.8)
Non-pegylated only	11 (10.3)	15 (13.9)
Pegylated only	5 (4.7)	1 (0.9)
Both	2 (1.9)	0
Median (range) spleen volume (cm^3^)	1202 (396–4631)	1307 (254–5147)

*BAT* best available therapy, *IFN* interferon

^a^Allele burden (defined as the ratio of mutant to wild-type *JAK2* p.V617F in hematopoietic cells) was reported among patients who were *JAK2* p.V617F mutation-positive at baseline

Among patients with extensive mutational analyses (*n* = 151), the percentage with ≥2 mutations in the 24 genes tested at baseline was 36.5% (27/74) in the ruxolitinib arm and 36.4% (28/77) in the BAT arm.

### Change from baseline in *JAK2* p.V617F allele burden

**Fig. 1:**
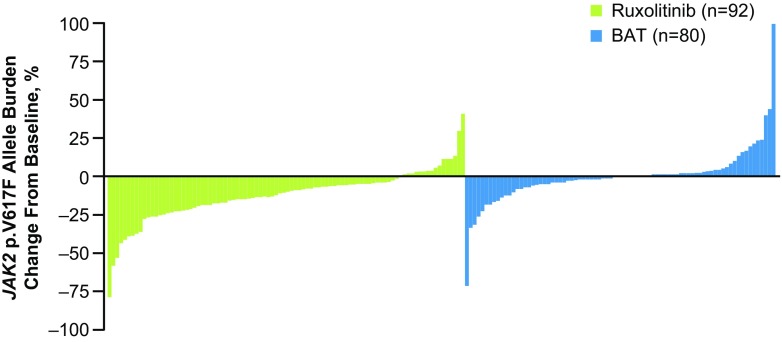
Change from baseline in *JAK2* p.V617F allele burden at week 32 in patients randomized to ruxolitinib and to BAT. *BAT* best available therapy

The percentage change from baseline in *JAK2* p.V617F allele burden at week 32 for individual patients is detailed in Fig. 1. Three patients in the ruxolitinib arm and one patient who received hydroxyurea in the BAT arm had a ≥50% reduction in allele burden at week 32.

**Fig. 2:**
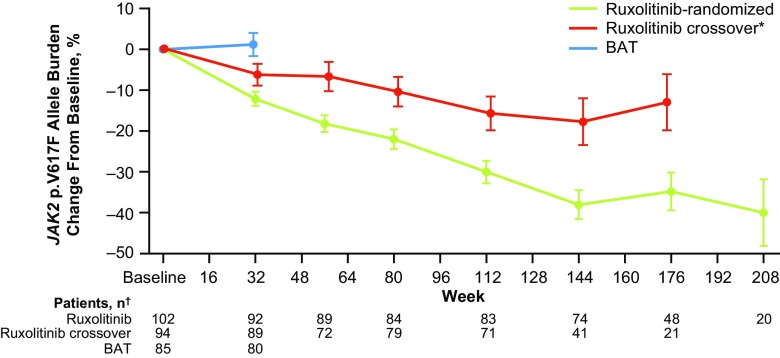
Mean change from baseline in *JAK2* p.V617F allele burden with long-term treatment in patients randomized to ruxolitinib and to BAT before and after crossover. *: In the ruxolitinib crossover arm, baseline allele burden was defined as the final assessment before crossing over from BAT to ruxolitinib. †: If there were <5 data points at a visit within a treatment group, data were excluded from the figure. *BAT* best available therapy

Overall, patients randomized to ruxolitinib had consistent allele burden reductions from baseline to week 208 (mean percent change, −40.0%; Fig. 2). The mean percentage change from baseline at week 32 in the BAT arm was 1.2%. Allele burden declined consistently over time for patients who crossed over to ruxolitinib (mean percent change, −13.0% at week 176).

**Fig. 3:**
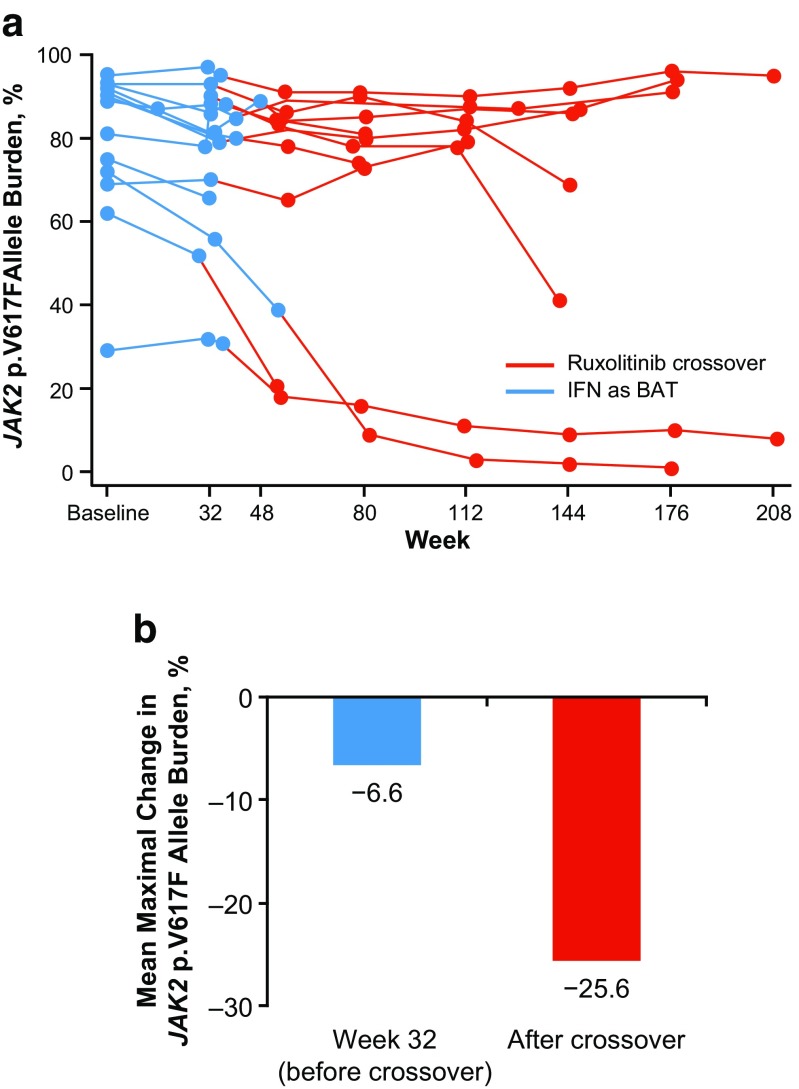
*JAK2* p.V617F allele burden before and after crossover in patients initially treated with IFN as BAT (*n* = 13). **a** Percentage change in *JAK2* p.V617F allele burden over time for individual patients. **b** Mean maximal percentage change in *JAK2* p.V617F allele burden before versus after crossover. *BAT* best available therapy, *IFN* interferon

The mean *JAK2* p.V617F allele burden at baseline was similar between patients who did and did not have prior IFN treatment (ruxolitinib, 72.1 vs 74.5%; BAT, 88.2 vs 72.0%, respectively). All 13 patients originally randomized to IFN as BAT crossed over to ruxolitinib. After crossover, 8 experienced allele burden decreases, 3 experienced allele burden increases, 1 had no change, and 1 did not have data available (Fig. 3a). Among patients treated with IFN as BAT, the mean maximal reduction in allele burden from baseline after crossover was 25.6% compared with 6.6% before crossover (Fig. 3b).

**Fig. 4:**
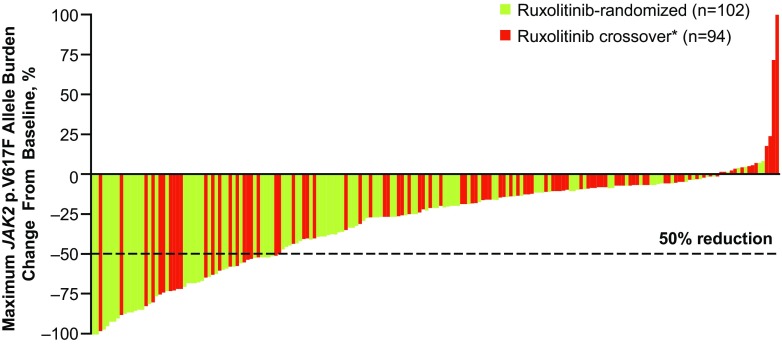
Maximum percentage change from baseline in *JAK2* p.V617F allele burden in patients in the ruxolitinib-randomized and ruxolitinib crossover groups. The *dotted line* represents a 50% reduction in *JAK2* p.V617F allele burden from baseline. *: In the ruxolitinib crossover arm, baseline allele burden was defined as the final assessment before crossing over from BAT to ruxolitinib. *BAT* best available therapy

The mean (SD) maximal reductions in allele burden in the ruxolitinib-randomized and crossover arms were −35.9% (29.7%) and −21.2% (30.7%), respectively; the median times to maximal reduction of allele burden were 25.9 and 18.2 months. The maximum percentage change from baseline in allele burden for individual patients is summarized in Fig. 4.

**Fig. 5:**
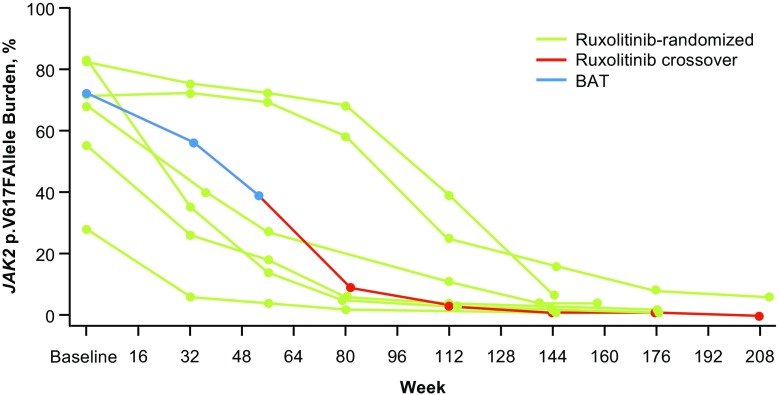
Patients with ≥90% reduction from baseline in *JAK2* p.V617F allele burden. Six ruxolitinib-randomized patients and one ruxolitinib crossover patient (*blue*, before ruxolitinib crossover; *red*, after crossover) had reductions in *JAK2* p.V617F allele burden of ≥90% from baseline. *BAT* best available therapy

Seven patients had a ≥90% reduction from baseline in *JAK2* p.V617F allele burden (ruxolitinib arm, *n* = 6; ruxolitinib crossover arm, *n* = 1; Fig. 5).

### Molecular response

Three patients who received ruxolitinib (ruxolitinib-randomized, *n* = 2, at 142.6 and 144.1 weeks; ruxolitinib crossover, *n* = 1, at 123.0 weeks) had a CMR; no patients experienced a CMR while receiving BAT (Table 2). A PMR was observed in 33 ruxolitinib-randomized patients and 1 patient randomized to BAT; an additional 20 patients experienced a PMR after crossover to ruxolitinib. Median times to PMR were 112.0 and 91.9 weeks in the ruxolitinib-randomized and ruxolitinib crossover arms, respectively; time to PMR was 32.0 weeks in the 1 BAT patient. Among patients who experienced a CMR or PMR and had evaluable mutation data, the most common non-*JAK2* mutations at baseline were in the *ASXL1* and *TET2* genes (Table 3).

**Table 2:** Molecular response (IWG-MRT/ELN criteria)^a^

	Ruxolitinib-randomized (*n* = 102)	Ruxolitinib crossover (*n* = 94)	BAT (*n* = 85)
CMR, *n* (%)	2 (2.0)	1 (1.1)	0
Median time to CMR (weeks)	143.4	123.0	N/A
PMR, *n* (%)	33 (32.4)	20 (21.3)	1 (1.2)
Median time to PMR (weeks)	112.0	91.9	32.0

*BAT* best available therapy, *CMR* complete molecular response, *IWG-MRT/ELN* International Working Group–Myeloproliferative Neoplasms Research and Treatment/European LeukemiaNet, *N/A* not applicable, *PMR* partial molecular response

^a^Among patients with a positive *JAK2* p.V617F mutation at baseline and ≥1 postbaseline assessment of allele burden

**Table 3:** Baseline mutation status in patients with molecular response

	Ruxolitinib-randomized			Ruxolitinib crossover		
Mutation^a^, *n* (%)	CMR (*n* = 2)	PMR (*n* = 24)	NMR (*n* = 49)	CMR (*n* = 1)	PMR (*n* = 16)	NMR (*n* = 50)
*ASXL1*	1 (50.0)	2 (8.3)	1 (2.0)	0	0	5 (10.0)
*TET2*	1 (50.0)	3 (12.5)	11 (22.4)	1 (100)	2 (12.5)	10 (20.0)
*JAK2* (other than *JAK2* p.V617F)	0	3 (12.5)	1 (2.0)	0	0	2 (4.0)
*JAK3*	0	1 (4.2)	0	0	0	0
*SOCS1*	0	1 (4.2)	1 (2.0)	0	0	1 (2.0)
*STAT5A*	0	0	1 (2.0)	0	1 (6.3)	1 (2.0)

*CMR* complete molecular response, *NMR* no molecular response, *PMR* partial molecular response

^a^No mutations were identified in the following genes among patients with a molecular response: *CALR*, *CBL*, *DNMT3A*, *EZH2*, *IDH1*, *IDH2*, *JAK1*, *NFE2*, *SF3B1*, *SH2B3*, *SOCS2*, *SOCS3*, *SRSF2*, *STAT1*, *STAT3*, *STAT5B*, and *U2AF1*

### Clinical outcomes

In the ruxolitinib-randomized and crossover groups, approximately 80% of patients with an allele burden reduction from baseline to last available observation of ≥20% also had a ≥35% reduction from baseline to last observation in spleen volume. However, <40% of patients whose allele burden was reduced by <20% also had a ≥35% reduction in spleen volume (Table 4).

**Table 4:** Final percentage reduction from baseline in *JAK2* p.V617F allele burden and spleen volume

	Ruxolitinib-randomized		Ruxolitinib crossover	
*JAK2* p.V617F allele burden reduction	≥20% (*n* = 52)	<20% (*n* = 47)	≥20% (*n* = 32)	<20% (*n* = 60)
Spleen volume reduction, *n* (%)				
≥35%	45 (86.5)	18 (38.3)	25 (78.1)	20 (33.3)
<35%	7 (13.5)	29 (61.7)	7 (21.9)	40 (66.7)

Only patients with baseline and postbaseline values for both spleen volume and allele burden were included

**Fig. 6:**
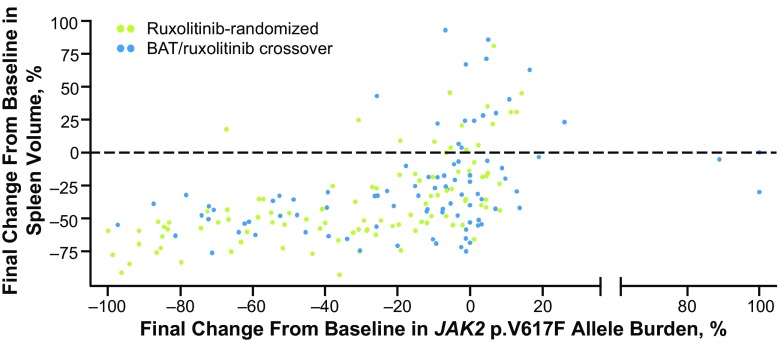
Final percentage change from baseline: spleen volume versus *JAK2* p.V617F allele burden in patients randomized to ruxolitinib versus those randomized to BAT who later crossed over to ruxolitinib. Each *dot* represents an individual patient. *BAT* best available therapy

No strong relationship was observed between the reduction of allele burden and the reduction of spleen volume from baseline to last available observation (Fig. 6).

No correlations were observed between allele burden changes and changes in select laboratory parameters (Hct levels, white blood cell counts, and platelet counts; Supplementary Fig. 1).

## Discussion

This post hoc analysis of allele burden data from the RESPONSE trial showed that patients who received ruxolitinib had greater reductions in allele burden than patients who received BAT. The degree to which ruxolitinib-associated reductions in *JAK2* p.V617F allele burden may correspond with improved clinical outcomes remains unclear. Patients treated with ruxolitinib who had more pronounced allele burden reductions (i.e., ≥20 vs <20% reduction from baseline to last observation) generally experienced greater spleen volume reductions; however, further studies are required to confirm this finding. Relationships between *JAK2* p.V617F allele burden changes and hematologic parameters of disease control, such as Hct levels, white blood cell counts, and platelet levels, were not observed.

Previous studies of PEG IFN-α2a found that many patients with PV who achieved a molecular response with PEG IFN-α2a also experienced reductions in *JAK2* p.V617F allele burden after 6 months of treatment [8–10]. In the current analysis, patients experienced molecular responses irrespective of when ruxolitinib was initiated during the disease course or whether there had been previous therapy with IFN.

Although the use of phase 3, randomized data for allele burden evaluations in patients with PV is novel, it is important to underscore that the strength of evidence for this post hoc analysis is limited. Furthermore, blood samples collected to evaluate allele burden were not intended for evaluations of molecular response or correlations with clinical outcomes.

Collectively, the data from this exploratory analysis suggest that ruxolitinib treatment (in randomized and crossover treatment arms) for up to 4 years provided progressive reductions in *JAK2* p.V617F allele burden in patients with PV who were resistant to or intolerant of hydroxyurea. Further research will be required to determine whether ruxolitinib-induced reductions in *JAK2* p.V617F allele burden correlate with clinically relevant improvements in patient outcomes.

## Electronic supplementary material

ESM 1 (PDF 383 kb)

## References

[1] Tefferi A, Vardiman JW (2008). Classification and diagnosis of myeloproliferative neoplasms: the 2008 World Health Organization criteria and point-of-care diagnostic algorithms. Leukemia.

[2] Tefferi A, Rumi E, Finazzi G, Gisslinger H, Vannucchi AM, Rodeghiero F, Randi ML, Vaidya R, Cazzola M, Rambaldi A, Gisslinger B, Pieri L, Ruggeri M, Bertozzi I, Sulai NH, Casetti I, Carobbio A, Jeryczynski G, Larson DR, Müllauer L, Pardanani A, Thiele J, Passamonti F, Barbui T (2013). Survival and prognosis among 1545 patients with contemporary polycythemia vera: an international study. Leukemia.

[3] Vannucchi AM, Antonioli E, Guglielmelli P, Longo G, Pancrazzi A, Ponziani V, Bogani C, Ferrini PR, Rambaldi A, Guerini V, Bosi A, Barbui T, MPD Research Consortium (2007). Prospective identification of high-risk polycythemia vera patients based on JAK2(V617F) allele burden. Leukemia.

[4] Vannucchi AM, Kiladjian JJ, Griesshammer M, Masszi T, Durrant S, Passamonti F, Harrison CN, Pane F, Zachee P, Mesa R, He S, Jones MM, Garrett W, Li J, Pirron U, Habr D, Verstovsek S (2015). Ruxolitinib versus standard therapy for the treatment of polycythemia vera. N Engl J Med.

[5] Verstovsek S, Vannucchi AM, Griesshammer M, Masszi T, Durrant S, Passamonti F, Harrison CN, Pane F, Zachee P, Kirito K, Besses C, Hino M, Moiraghi B, Miller CB, Cazzola M, Rosti V, Blau I, Mesa R, Jones MM, Zhen H, Li J, Francillard N, Habr D, Kiladjian J-J (2016). Ruxolitinib versus best available therapy in patients with polycythemia vera: 80 week follow-up from the RESPONSE trial. Haematologica.

[6] Collier P, Patel K, Waeltz P, Rupar M, Luthra R, Liu PC, Hollis G, Huber R, Verstovsek S, Burn TC (2013). Validation of standards for quantitative assessment of JAK2 c.1849G>T (p.V617F) allele burden analysis in clinical samples. Genet Test Mol Biomarkers.

[7] Barosi G, Mesa R, Finazzi G, Harrison C, Kiladjian JJ, Lengfelder E, McMullin MF, Passamonti F, Vannucchi AM, Besses C, Gisslinger H, Samuelsson J, Verstovsek S, Hoffman R, Pardanani A, Cervantes F, Tefferi A, Barbui T (2013). Revised response criteria for polycythemia vera and essential thrombocythemia: an ELN and IWG-MRT consensus project. Blood.

[8] Quintás-Cardama A, Kantarjian H, Manshouri T, Luthra R, Estrov Z, Pierce S, Richie MA, Borthakur G, Konopleva M, Cortes J, Verstovsek S (2009). Pegylated interferon alfa-2a yields high rates of hematologic and molecular response in patients with advanced essential thrombocythemia and polycythemia vera. J Clin Oncol.

[9] Kiladjian JJ, Cassinat B, Chevret S, Turlure P, Cambier N, Roussel M, Bellucci S, Grandchamp B, Chomienne C, Fenaux P (2008). Pegylated interferon-alfa-2a induces complete hematologic and molecular responses with low toxicity in polycythemia vera. Blood.

[10] Kiladjian JJ, Giraudier S, Cassinat B (2016). Interferon-alpha for the therapy of myeloproliferative neoplasms: targeting the malignant clone. Leukemia.

