# Supplementary material for: Ruxolitinib reduces JAK2 p.V617F allele burden in patients with polycythemia vera enrolled in the RESPONSE study
Source: Ann Hematol. 2017 Apr 30;96(7):1113–20. doi: 10.1007/s00277-017-2994-x (PMC5486779; doi:10.1007/s00277-017-2994-x)
Supplement: Supplementary file 1 — (PDF 383 kb) [file 277_2017_2994_MOESM1_ESM.pdf]

Ruxolitinib Reduces *JAK2* p.V617F Allele Burden in Patients With Polycythemia Vera Enrolled in the RESPONSE Study

Alessandro Maria Vannucchi, MD,[1] Srdan Verstovsek, MD, PhD,[2]
Paola Guglielmelli, MD, PhD,[1] Martin Griesshammer, MD,[3] Timothy C. Burn, PhD,[4]
Ahmad Naim, MD,[4] Dilan Paranagama, PhD,[4] Mahtab Marker, PhD,[5]
Brian Gadbaw, MD,[5] Jean-Jacques Kiladjian, MD, PhD[6]

[1]CRIMM, Center for Research and Innovation of Myeloproliferative Neoplasms, AOU Careggi, Laboratorio Congiunto and Department of Experimental and Clinical Medicine, University of Florence, Florence, Italy; [2]The University of Texas MD Anderson Cancer Center, Houston, TX, USA; [3]Johannes Wesling University Clinic, Minden, Germany; [4]Incyte Corporation, Wilmington, DE, USA; [5]Novartis Pharmaceuticals Corporation, East Hanover, NJ, USA; [6]Hôpital Saint-Louis, Paris, France

Supplementary Material

Supplementary Fig. 1 No correlations were observed between changes in *JAK2* p.V617F allele burden and Hct **(a)**, white blood cell count **(b)**, or platelet level **(c)** in patients randomized to ruxolitinib treatment compared with those randomized to BAT who later crossed over to ruxolitinib. BAT, best available therapy; Hct, hematocrit.

Note: Includes crossover patients who had positive allele burden at baseline (final observation before crossover).

Supplementary Fig. 1

a

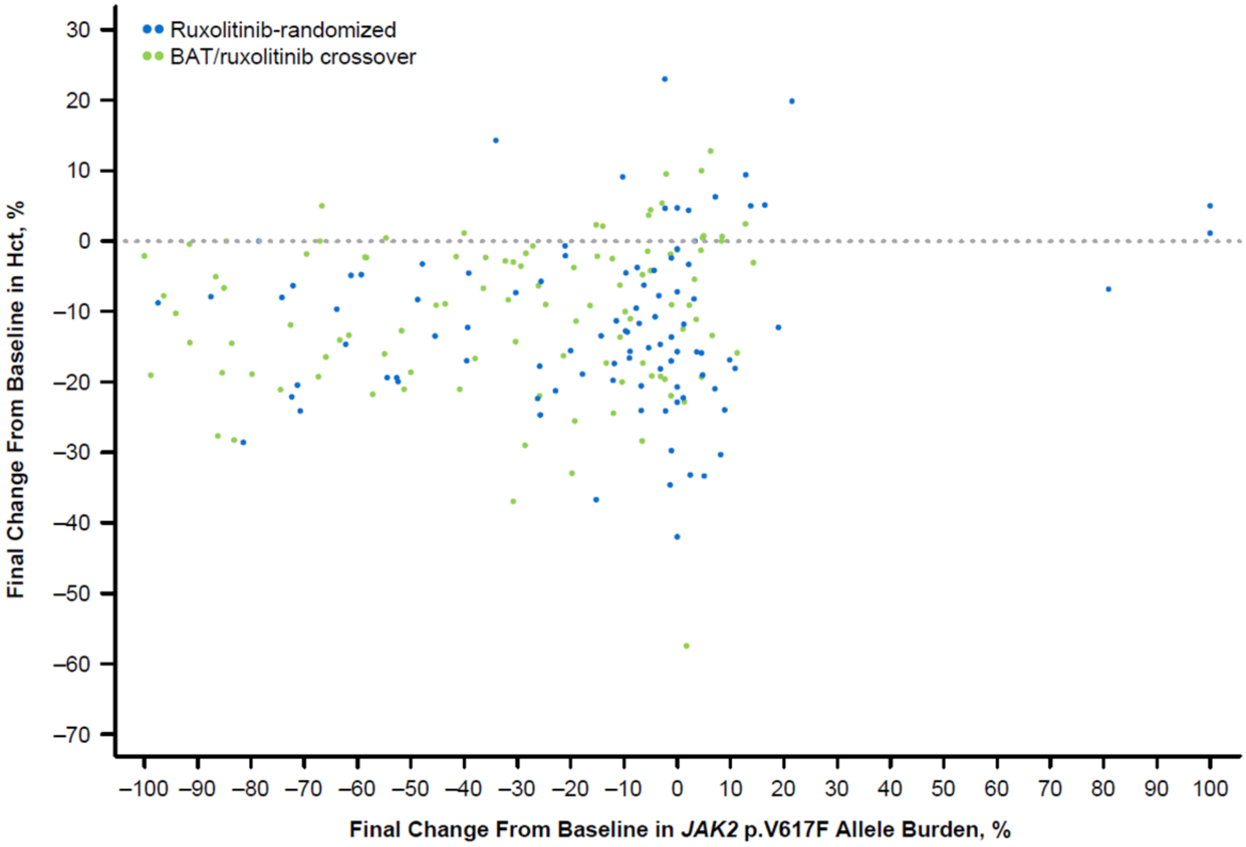

b

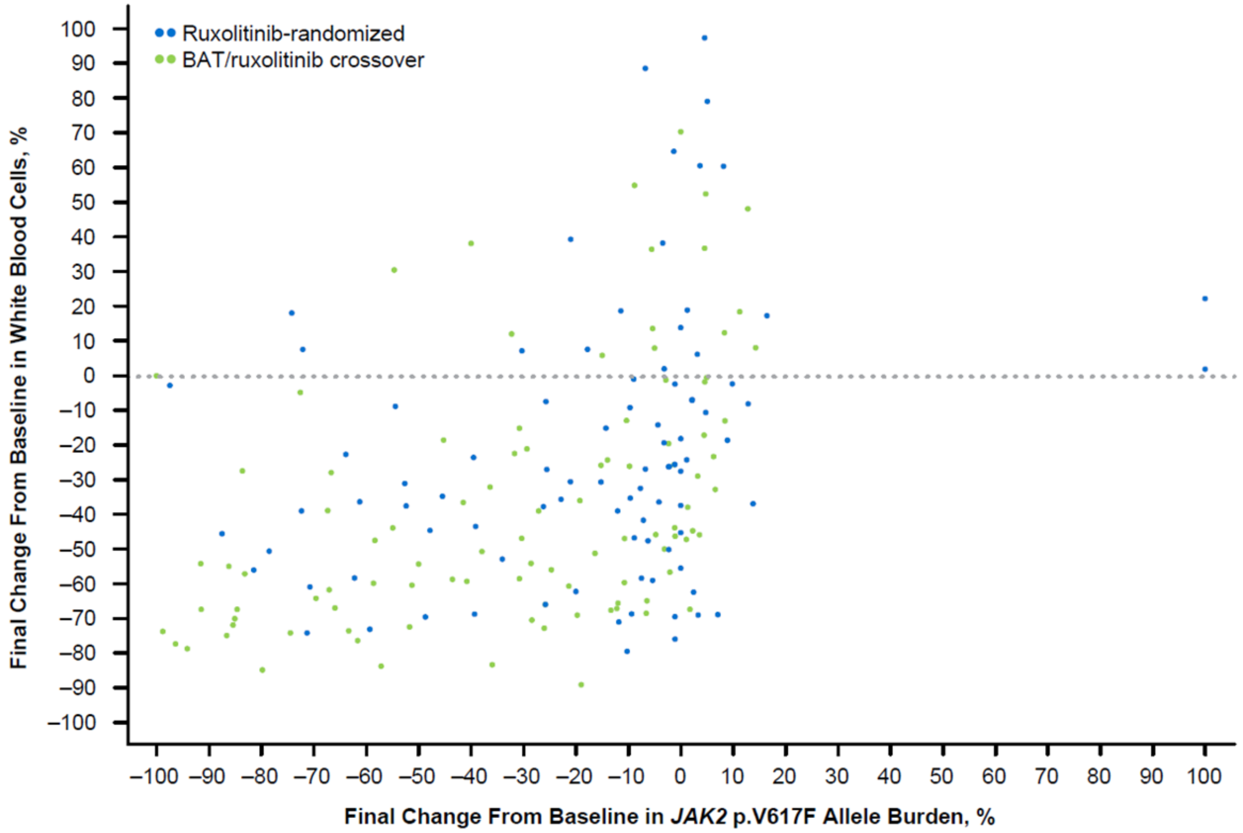
Ruxolitinib-randomized
BAT/ruxolitinib crossover
Final Change From Baseline in White Blood Cells, %
100
90
80
70
60
50
40
30
20
10
0
−10
−20
−30
−40
−50
−60
−70
−80
−90
−100
−100 −90 −80 −70 −60 −50 −40 −30 −20 −10 0 10 20 30 40 50 60 70 80 90 100
Final Change From Baseline in JAK2 p.V617F Allele Burden, %

c

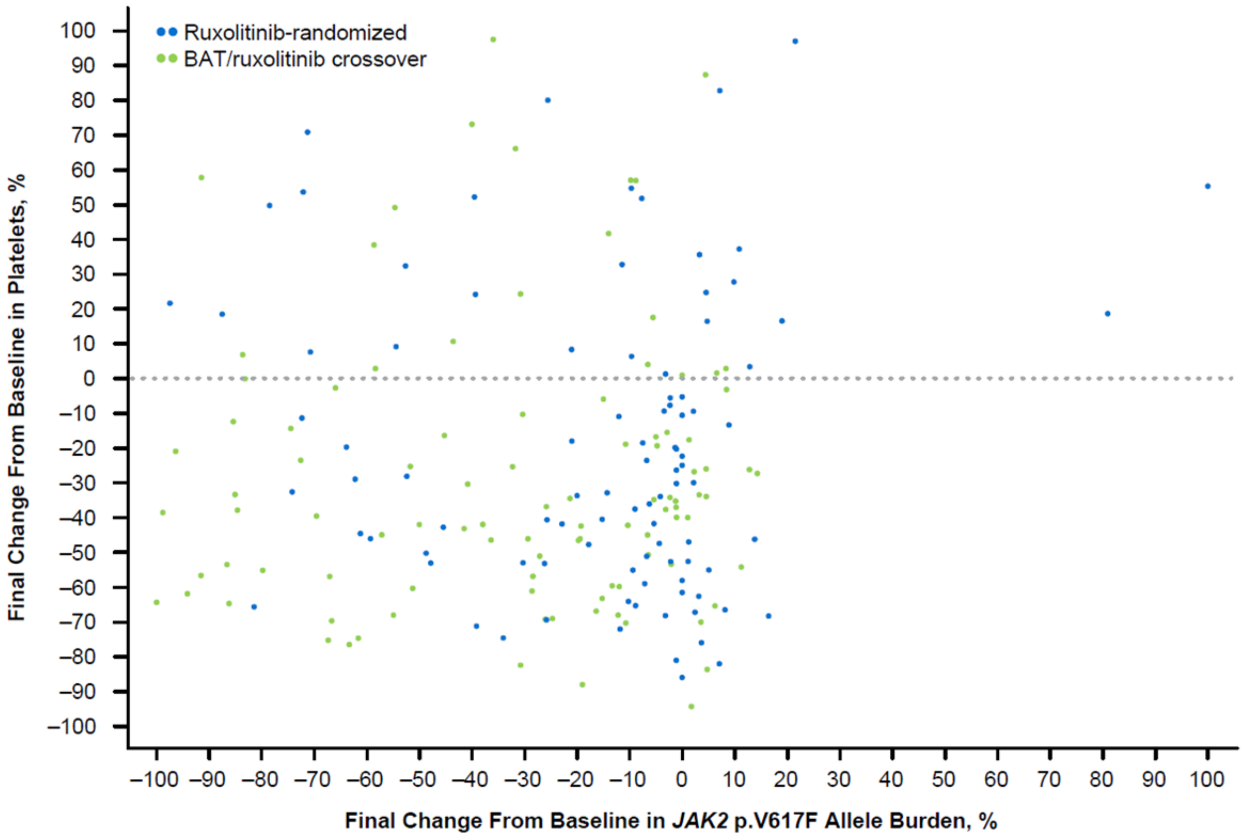
Ruxolitinib-randomized
BAT/ruxolitinib crossover
Final Change From Baseline in Platelets, %
100
90
80
70
60
50
40
30
20
10
0
−10
−20
−30
−40
−50
−60
−70
−80
−90
−100
−100 −90 −80 −70 −60 −50 −40 −30 −20 −10 0 10 20 30 40 50 60 70 80 90 100
Final Change From Baseline in JAK2 p.V617F Allele Burden, %
